# Does Cosmetic Rhinoplasty Affect Nose Function?

**DOI:** 10.5402/2011/615047

**Published:** 2011-09-18

**Authors:** Seyed Behzad Pousti, Sam Touisserkani, Maryam Jalessi, Seyed Kamran Kamrava, Nader Sadigh, Ashkan Heshmatzade Behzadi, Arash Arvin

**Affiliations:** ENT-Head and Neck Research Center, Hazrat Rasoul Akram Hospital, Tehran University of Medical Sciences, Tehran 14455-364, Iran

## Abstract

*Objective*. To evaluate the changes in nasal dimensions of healthy Iranian volunteered for cosmetic rhinoplasty after surgery using acoustic rhinometry. *Methods*. Pre- and postoperative nasal dimension of 36 cases undergoing cosmetic rhinoplasty were compared using acoustic rhinometry (AR), and the measured variables were distance to first and second constriction (d1, d2), first and second minimal cross-sectional area (MCA1, 2), and volume. *Results*. Mean age (SD) of cases were 24.63 (4.4) years. Septoplasty was performed in 12 cases (33.3%). After surgery, bilateral d1 and both MCA2 decreased significantly, while significant increase was observed in MCA1 postoperatively using decongestant. Cases with septoplasty experienced more increase in MCA1 and less constriction in MCA2 postoperatively. In cases with rhinoplasty alone, they received benefit from double osteotomy in MCA1. In either group of rhinoplasty with and without septoplasty, placing a strut was beneficial for patients. *Discussion*. The cross-sectional area of the nose is a major factor in the determination of airflow. Cosmetic rhinoplasty may generate a mix effect on nose function. Performing osteotomy may better help patients to save nasal patency, septoplasty is beneficial even in mildly deviated septums, and placing a strut may be beneficial in most of the cases.

## 1. Introduction

Modern nasal surgery takes into account the cosmetic appearance of the nose but also seeks to improve the natural functions of the nose. Rhinosurgeons emphasize the functional aspect of septorhinoplasty. So far, only a few publications with exact data exist [1].

Furthermore, in modern respiratory medicine, objective measurement of airway patency is a must. Measurement of nasal cavity geometry has proven to be a great challenge for researchers in modern rhinology [2]. Functioning of the human nose is greatly dependent on nasal cavity geometry. The most common method used in these measurements is acoustic rhinometry (AR), which is based on analysis of reflected acoustic impulses [3, 4]. It has demonstrated that the cross-sectional area of the nasal airway measured by acoustic rhinometry and the area found by calculations using data from rhinomanometry has close correlation [3, 5]. Furthermore, several significant associations between nasal cavity dimensions and nasal airflow have been reported [6]. And the most important benefit of acoustic rhinomanometry is that is easy to perform and requires less cooperation from subject [7].

Few studies addressed the nose function after cosmetic rhinoplasty [8–11]. Most of these studies were performed in patients with abnormal nose function before surgery. However, nose geometry may be influenced by various factors including race, age, sex, height, weight, and smoking habit. Race is the most important factor [12, 13].

In this paper we evaluate the changes in nasal dimensions of healthy Iranian volunteered for cosmetic rhinoplasty after surgery using acoustic rhinometry and comparing differences in length, minimal cross sectional area (MCA), and volume of each nasal cavity before and after surgery. In addition, factors that may influence these changes would be studied.

## 2. Method

Seventy two nasal cavities were analyzed from 36 cases undergoing cosmetic rhinoplasty. The cases were all healthy, without any prior history of nose surgery, fracture, allergy or asthma, and sinus disorders, and they had no complaint about nasal obstruction. All cases were Iranian aged at least 18 who were visited in ENT clinic of Rassul-e-Akram teaching hospital between 2006 and 2008.

The surgeries according to the structure of the nose were open rhinoplasty with or without septoplasty. T-type of osteotomy (single versus double), placement of columellar strut, or other variations of surgery were dependent on the surgeons preference. All operations were performed by a single experienced surgeon, and septoplasty was performed in the case of moderate-to-severe septal deviation. 

Tests were conducted using an Eccovision Acoustic Rhinometer (HOOD Laboratories). The device was consists of a sound source (loudspeaker) distally positioned in relation to a 24 cm tube equipped with a microphone for acquisition in its proximal portion. A sound pulse was generated with a peak power of 146 dB sound pressure level and a 50-ms duration.

All measurements were performed after a short period of acclimatization and in a relatively quiet room at normal temperature (mean = 21.4°C) to minimize artifacts from physical stress, environmental noise, and temperature changes.

The measurements were performed during a breathing pause while patients were in a sitting position. The nosepiece used in the measurements was 5 cm in length and was anatomically sculptured. To ensure a tight connection between the nosepiece and tip of the nose, a small amount of ultrasound transmission gel was applied to the edge of the nosepiece. Care was taken not to obstruct the nasal vestibule with gel or deform the nose during testing. The angle of the incident acoustic impulse was about 45° with respect to a line joining the base of the piriform aperture of the nose to the tragus. Measurement performed in either of two conditions of without a decongestant and with a decongestant, respectively. 

The first measurement performed before surgery and the second measurement performed 3 months after surgery. Each measurement repeated 3 times and average value was recorded. 

The technique we used allowed us to analyze two local minimum points giving us minimum cross-sectional areas (MCA1 and MCA2) and distance (d1 and d2) to the points from the nostril and volumes of nostrils.

To examine the geometrically difference in pre-and postoperative nasal cavities, paired *T* test used for repeated measurements.

## 3. Results

The data of 72 nasal cavities were analyzed. Mean age (SD) of cases were 24.63 (4.4) years, minimum age was 18, and maximum age was 40. Male-to-female ratio was 1 : 3. None cases had complaints of breathing problems before and after the surgery, and all of our cases were satisfied with their surgery result. Septoplasty was performed in 12 cases (33.3%). Mean (SD) geometric values of cases preoperatively with and without decongestant are demonstrated in Table 1. By the pair wise comparison of cases without decongestant before and after surgery, right (R) d1, bilateral d2, and left (L) MCA2 did not change significantly after surgery. L d1, R MCA2, and bilateral volume (V) decreased significantly. Furthermore, bilateral MCA1 increased significantly. However, pairwise comparison of nasal dimensions with decongestant revealed that bilateral d2 and V did not changed significantly postoperatively. Bilateral d1 and both MCA2 decreased significantly, while significant increase was observed in MCA1 postoperatively (Table 2).

In order to assess the result of septoplasty, patients divided into 2 groups of rhinoplasty plus septoplasty and rhinoplasty alone. Comparison of the finding showed that cases with septoplasty would experience more increase in MCA1 (increase in MCA1 was significant and much more in decongested state of group with septoplasty) and less constriction in MCA2 postoperatively (significant constriction were observed in both groups. However, amount of this constriction was more in group without septoplasty) (Table 3).

In cases of rhinoplasty without septoplasty, increase in MCA1 was significant only in group of single osteotomy without decongestant. Although changes in MCV2 were significant in both with and without decongestion groups of double osteotomy, but the net effects were not clinically significant comparing single osteotomies.  Table 4 demonstrates that cases with rhinoplasty alone would benefit from double osteotomy in MCA1.

Effect of osteotomy on patency of nose in cases with septoplasty is shown in Table 5; it is more prominent at the level of MCA1.

Placing a columellar strut in group of cases of rhinoplasty plus septoplasty has significant effect both at MCA1 and MCA2 levels (Table 6). In cases of rhinoplasty alone, placing a columellar strut caused greater amount of increase in MCA1. MCA2 after decongestion significantly increased in group without columellar strut, but it did not changed in group with replacing a columellar strut.

Regression analysis of difference after surgery in MCA1, MCA2, and V revealed no fix significant effect for septoplasty, osteotomy, and placing a columellar strut.

## 4. Discussion

Rhinomanometry and acoustic rhinometry provide the best methods for objective assessment of nasal obstruction. Advanced equipment for these methods is now available, and most devices are reliable provided that care is taken to calibrate the device properly. In the current study, we used an AR to measure the dimensions of the nose [14]. 

AR evaluation of the nasal airway has been reported to be accurate when compared with MRI2 and CT [15, 16]. The closest correlations have been noted after decongestant use and in the most anterior 5 cm of the nasal cavity [16]. It is believed that acoustic rhinometry provided less variability of results than those obtained with rhinomanometry [3]. In the study of Corey he concludes that the anatomic correlate of CSA1 (cross-sectional area) is not as clearly delineated; however, it likely corresponds to an area at or near the nasal valve. Furthermore, CSA2 correspond to the general areas of the anterior portions of the inferior turbinate. He claimed that in conjunction with a careful clinical exam, AR can provide objective documentation and diagnosis for better treatment of nasal airway blockage [17]. 

Acoustic rhinometry also seems very suitable for evaluation of the nasal cavity in cases where septoplasty and turbinoplasty is considered as well as for the postoperative objective evaluation [18]. Haarmann et al. studied the changes in nasal airways after Le Fort I osteotomy and functional rhinosurgery with both AR and rhinomanometry techniques. He declare that rhinomanometry and acoustic rhinometry are reliable and objective methods of determining functional and geometric changes in the nasal cavity after Le Fort I osteotomy [19].

In this paper we described Reference values in normal and decongested noses of normal Iranian population with acoustic rhinometry. All of our cases were normal people without significant symptoms of nasal dysfunction in their nose, and surgery was performed just because of aesthetic reasons.

Comparing our finding with Mohebbi et al. [13], in Iranian population, no significant difference was observed in MCAs and V. This finding supports the original idea of study to enroll cases with normal nose geometry. 

By comparing nose geometry before and after surgery in both normal state and decongested state, considering presence of inflammation after surgery and more precise estimation in decongested estate [20], the main findings were a reduction in d1, an increase in MCA1, a decrease in MCA2 and no change in V and d2. These findings are consistent with findings of Greymer that reported reduction in internal dimensions of the nasal cavity especially the anterior dimensions [21]. This finding highlight the fact that second constriction is a critical place during the surgery and adequate attention should be paid to this point. The anatomic correlate of this point is anterior portion of the inferior turbinate [20], hence decreasing the resistance in this region could be beneficial for patients.

Operations on the nasal septum are frequently performed with the intention of improving the nasal airway. For this reason, they form much of the routine workload in most otolaryngology departments. A meta-analysis performed by Singh et al. demonstrated that an overall significant improvement following surgery is present in patient undergoing septal surgery [22]. 

In our study, comparison of cases of rhinoplasty with and without septoplasty revealed that the cases with septoplasty would experience more increase in MCA1 and less constriction in MCA2. As a conclusion, even in the cases which mild septal deviation is present, performing the septoplasty may increase patient nose patency.

Osteotomy as a major procedure in rhinoplasty was considered in analysis. More increase in MCA1 and no significant effect on MCA2 were observed in patients in double osteotomy group comparing group of single osteotomy. It seems as if double osteotomy positively affects nose function by more increase in MCA1. Double osteotomy is positively affecting MCA1 considering the cases without septoplasty or cases with septoplasty.

In either group of rhinoplasty with and without septoplasty, placing a strut was beneficial for patients.

In the study of Oeken and Kiefer on 52 cases of septorhinoplasty total nasal airflow did not changed postoperatirely. And if only the worse nasal side was considered rhinometry showed a highly significant improvement [1]. It should be mentioned that in the above study, all cases suffered from a deviated septum and some degree of obstruction.

Anselmo-Lima et al. in a study of 30 cases of rhinoplasty with lateral osteotomy observed that there is a reduction in nasal valve area (significant reduction in MCA1) as well as in anterior volume of the nose [23]. They propose more criteria to indicate rhinoplasty, because the procedure might lead to nasal obstruction.

In the study of Kemker et al. on 31 cases of septoplasty and sinonasal surgical procedures, patients in the septoplasty-only group showed a statistically significant increase in volume as measured by AR. Patients who had septoplasty plus other sinonasal procedures showed significant increases in volume and cross-sectional area (CSA) 3, whereas CSAs 1 and 2 increased also, but not significantly [24].

Shemen and Hamburg studied 24 patients undergone septal surgery, and Skouras et al. studied 16 patients, the improvement in nasal patency was seen in both studies. However, cases in both studies have complained of nasal obstruction before surgery [25, 26].

In a study by Can et al. on 26 children with septal deviation, septoplasty was performed, and MCA and V were measured by AR. The results showed that the surgery was successful; however, They claim that surgery should be restricted to only the pathologic area and should be conservative [18].

## 5. Limitation

As we know, AR has some limitations, as it cannot measure the Columella width in nasal geometry and also differentiate the nostrils shape that has effect on the turbulence of air delivered to nasal cavity.

## 6. Conclusion

As we know, the cross-sectional area of the nose is a major factor in the determination of airflow, with airflow increasing as the cross-sectional area increases [8]. Hence, according to the above findings, cosmetic rhinoplasty may generate a mix effect on nose function. Comparison of our finding to other studies shows that condition of the patient before surgery is the principal factor for the prediction of surgery results. Putting all together, surgeons should be aware of common pattern of change in nose geometry; care must be taken in intervention in the region of second constriction. Performing osteotomy may better help patients to save nasal patency, septoplasty is beneficial even in mildly deviated septum, and placing a strut may be beneficial in most of the cases.

## Figures and Tables

**Table 1:** Mean value of nostril geometry before surgery.

	Mean (SD)	Mean (SD) with decongestant
L d1	3.22 (1.29)	3.38 (1.10)
R d1	3.33 (10.95)	3.48 (1.23)
L MCA1	.52 (.25)	.52 (.19)
R MCA1	.51 (.23)	.51 (.22)
L d2	2.90 (.77)	2.93 (.74)
R d2	2.77 (.73)	2.66 (.63)
L MCA2	.54 (.13)	.53 (.14)
R MCA2	.55 (.16)	.54 (.16)
L V	3.92 (.68)	4.05 (.63)
R V	4.10 (.83)	4.08 (.79)

**Table 2:** Difference in geometry of nostrils after surgery. Positive and negative values, respectively, demonstrate decrease and increase in dimensions.

	Mean (SD) difference	Mean (SD) difference with decongestant
Difference in L d1	1.27 (1.65)*	1.55 (2.09)*
Difference in R d1	3.43 (10.68)	1.35 (2.25)*
Difference in L MCA1	−.25 (.44)*	−.27 (.43)*
Difference in R MCA1	−.21 (.46)*	−.24 (.41)*
Difference in L d2	.05 (.90)	−.13 (.95)
Difference in R d2	−.048 (1.07)	−.27 (87)
Difference in L MCA2	.062 (.45)	.12 (.18)*
Difference in R MCA2	.145 (.16)*	.11 (.18)*
Difference in L V	.349 (.93)*	.16 (1.02)
Difference in R V	.351 (1.02)*	−.005 (1.04)

*Significant difference (*P* < 0.05).

**Table 3:** Difference in geometry of nostrils after surgery in cases underwent rhinoplasty alone and cases with rhinoplasty and septoplasty.

Mean difference	Rhinoplasty		Rhinoplasty plus septoplasty	
	Without decongestant	With decongestant	Without decongestant	With decongestant
Left d1	1.52*	1.13	1.15*	1.75*
Right d1	1.98*	1.10	4.15	1.47*
Left MCA1	−.37*	−.01	−.20	−.39*
Right MCA1	−.41*	−.08	−.11	−.32*
Left d2	.21	−.13	−.02	−.13
Right d2	−.35	−.16	.10	−.33
Left MCA2	.15*	.18*	.01	.09*
Right MCA2	.17*	.12	.13*	.10*
Left V	.02	.64	.51*	−.06
Right V	.10	.50	.47*	−.24

*Significant difference (*P* < 0.05). Positive and negative values, respectively, demonstrate decrease and increase in dimensions.

**Table 4:** Effect of osteotomy on geometry of nose in cases without septoplasty.

Mean difference	Double osteotomy *n* = 8		Single osteotomy *n* = 4	
	Without decongestant	With decongestant	Without decongestant	With decongestant
Left d1	1.39	1.89*	1.79	.19
Right d1	1.29	1.65	3.38*	.42
Left MCA1	−.48*	.09	−.15	−.14
Right MCA1	−.42*	.01	−.39	−.21
Left d2	.43	−.68	−.22	.55
Right d2	−.13	−.56	−.80	.32
Left MCA2	.16*	.17*	.13*	.20
Right MCA2	.17*	.20*	.18*	.03
Left V	−.29	.98	.65	.21
Right V	−.12	1.10	.56	−.25

*Significant difference (*P* < 0.05). Positive and negative values, respectively, demonstrate decrease and increase in dimensions.

**Table 5:** Effect of osteotomy on geometry of nose in cases with Rhinoplasty plus septoplasty.

Mean difference	Double osteotomy *n* = 5		Single osteotomy *n* = 4	
	Without decongestant	With decongestant	Without decongestant	With decongestant
Left d1	1.39	1.89*	1.79	.19
Right d1	1.29	1.65	3.38*	.42
Left MCA1	−.48*	.09	−.15	−.14
Right MCA1	−.42*	.01	−.39	−.21
Left d2	.43	−.68	−.22	.55
Right d2	−.13	−.56	−.80	.32
Left MCA2	.16*	.17*	.13*	.20
Right MCA2	.17*	.20*	.18*	.13
Left V	−.29	.98	.65	.21
Right V	−.12	1.10	.56	−.25

*Significant difference (*P *< 0.05). Positive and negative values, respectively, demonstrate decrease and increase in dimensions.

**Table 6:** Effect of placing strut on geometry of nose in cases with only rhinoplasty.

Mean difference	With Strut *n* = 11		Without strut *n *= 13	
	Without decongestant	With decongestant	Without decongestant	With decongestant
Left d1	2.07*	1.35	.37	1.98*
Right d1	1.42	.13	6.47	2.25*
Left MCA1	−.61*	−.38	.14*	−.39*
Right MCA1	−.30*	−.19	.05	−.40*
Left d2	−.02	.05	−.02	−.25
Right d2	.28	−.01	−.04	−.51
Left MCA2	−.06	−.02	.08	.16*
Right MCA2	.13*	.00	.12*	.16*
Left V	.17	−.72*	.79*	.32
Right V	−.07	−.65	.94*	−.00

*Significant difference (*P* < 0.05). Positive and negative values, respectively, demonstrate decrease and increase in dimensions.
